# Tuberculosis due to Resistant Haarlem Strain, Tunisia

**DOI:** 10.3201/eid1106.041365

**Published:** 2005-06

**Authors:** Helmi Mardassi, Amine Namouchi, Raja Haltiti, Mourad Zarrouk, Besma Mhenni, Anis Karboul, Neila Khabouchi, Nico C. Gey van Pittius, Elizabeth M. Streicher, Jean Rauzier, Brigitte Gicquel, Koussay Dellagi

**Affiliations:** * Institut Pasteur de Tunis, Tunis-Belvédère, Tunisia;; † Hôpital Régional de Menzel-Bourguiba, Menzel-Bourguiba, Tunisia;; ‡ University of Stellenbosch, Stellenbosch, South Africa;; § Institut Pasteur, Paris, France

**Keywords:** Mycobacterium tuberculosis, Multidrug-resistant, outbreak, Haarlem strain, IS6110 RFLP, Accelerated transmission, genotyping

## Abstract

Multidrug-resistant tuberculosis was diagnosed in 21 HIV-negative, nonhospitalized male patients residing in northern Tunisia. A detailed investigation showed accelerated transmission of a *Mycobacterium tuberculosis* clone of the Haarlem type in 90% of all patients. This finding highlights the epidemic potential of this prevalent genotype.

The ability of multidrug-resistant (MDR) strains of *Mycobacterium tuberculosis* to cause epidemics and spread globally contrasts with the initial perception that MDR tuberculosis (MDR-TB) has a reduced potential for transmission (1,2). In this respect, the W/Beijing type appears to be most common in humans and accounts for most reported MDR-TB outbreaks (3).

In this report, we provide evidence for the epidemic potential of another worldwide prevalent *M. tuberculosis* genotype, namely, the Haarlem family (4,5). The identified strain is MDR and has rapidly expanded within immunocompetent and nonhospitalized patients.

## The Study

*M. tuberculosis* isolates were obtained from the Laboratory of Mycobacteriology of the Institut Pasteur de Tunis as part of the National Tuberculosis Surveillance Program. All samples (884 specimens) from patients with suspected TB residing in northern Tunisia (Bizerte) from August 2001 to October 2003 were forwarded to us by the referral regional hospital. This hospital serves a region with 483,086 people and an area of 3,501 km^2^. The incidence of TB in this area from 2001 to 2002 was 29/100,000 male patients and 11/100,000 female patients. All patients received the standard chemotherapy regimen of the Tunisian National Tuberculosis Program, i.e., 2 months of rifampicin, isoniazid, pyrazinamide, and streptomycin, followed by 4 months of rifampicin and isoniazid (2RHZS/4RH). This regimen was introduced into the region in 1995. Of the 193 *M. tuberculosis* isolates recovered, 20 were MDR. The corresponding patients were interviewed, and detailed epidemiologic investigations were conducted according to described protocols (6). In April 2004, while the study was in progress, a new MDR case was diagnosed.

Analyses by IS*6110* restriction fragment length polymorphism (IS*6110* RFLP), ligation-mediated polymerase chain reaction (PCR), and spoligotyping were carried out by using standard protocols (7–9). Typing of the polymorphic GC-rich repetitive sequence (PGRS) with probe MTB484 (1) was conducted according to a previously reported protocol (10), with the exception that DNA was digested with *Alu*I. Isolates were assigned to principal genetic groups according to the polymorphisms in the *katG* and *gyrA* genes (11). The following primer pairs were used to sequence *rpoB*, *katG*, and *pncA* gene mutations that confer resistance to rifampicin, isoniazid, and pyrazinamide, respectively: *rpoB* (5´-ATCACACCGCAGACGTTG-3´, 5´-TGCATCACAGTGATGTAGTCG-3´); *katG* (5´- CGTCGAAACAGCGGCGCTGA-3´, 5´-CAAGCGCCAGCAGGGCTCTT-3´); and *pncA* (5´-GGCGCACACAATGATCGGTG-3´, 5´-GCTTTGCGGCGAGCGCTCCA-3´). The recently described single nucleotide polymorphisms in putative *M. tuberculosis* mutator genes *mutT1*, *mutT2*, *mutT3*, and *ogt* were investigated with the same protocol reported by Rad et al. (12). DNA sequencing was conducted directly on the purified PCR products by using the Prism Ready Reaction Dye Deoxy Terminator Cycle sequencing kit on an ABI Prism 377 DNA sequencer (Applied Biosystems, Foster City, CA, USA).

Epidemiologic and clinical data indicated that all patients with MDR-TB were male with a mean age of 31 years at diagnosis (Table 1). All were Tunisians and permanently resided in the northern part of the country (Bizerte). All patients were seronegative for HIV with no documented history of travel abroad, and none had a history of immunosuppression, diabetes, or respiratory diseases other than TB. Mapping of the 21 patients with MDR-TB according to their residence sites showed that they were mostly scattered over the northeastern part of the region (surface area ≈1,000 km^2^) with no concentration in a particular locality (data not shown). Resistance to 5 first-line drugs was observed for most isolates (Table 2).

**Table 1:** Clinical characteristics of 21 patients with multidrug-resistant tuberculosis (MDR-TB), Bizerte, Tunisia, 2001–2004*

Patient	Age (y)	Sex	Case history	Isolate used for molecular typing	Initial diagnosis of MDR-TB	Epidemiologic characteristic	Chest radiography
P1	24	M	PT	Follow-up, Oct 2001	Sep 2001	Brother of patient 14	Right apical cavity nodular lesion
P2	26	M	NC	Initial	Oct 2001	Same penitentiary as patients 7 and 9	Left mid-lung nodular opacity with excavation
P3	25	M	NC	Initial	Oct 2001	None apparent	Bi-apical nodular opacity
P4	62	M	NC	Initial	Nov 2001	None apparent	Right apical nodular opacity
P5	26	M	PT	Follow-up, Feb 2002	Sep 2000	Brother of patient 18	Diffuse nodular lesions and multiple cavities
P6	23	M	PT	Follow-up, Feb 2002	Feb 2001	None apparent	Right apical and median bilateral nodular opacity
P7	24	M	PT	Follow-up, Mar 2002	Sep 2000	Same penitentiary as patients 2 and 9	Right lobe apical nodular opacity
P8	27	M	NC	Initial	Jun 2002	None apparent	Right apical nodular opacity
P9	34	M	PT	Follow-up, Jun 2002	Aug 2001	Same penitentiary as patients 2 and 7	Right apical nodular opacity and left diffuse nodular opacity
P10	21	M	NC	Initial	Jun 2002	None apparent	Right lobe apical nodular opacity and cavity
P11	42	M	NC	Initial	Jul 2002	None apparent	Basal nodular opacity of the right and left lung
P12	23	M	NC	Initial	Jul 2002	None apparent	Left apical cavity and nodular opacity
P13	29	M	PT	Follow-up, Aug 2002	Sep 2001	None apparent	Bilateral apical and diffuse opacity
P14	34	M	NC	Initial	Nov.2002	Brother of patient 1	Left apical cavity and nodular lesion
P15	51	M	PT	Follow-up, Nov 2002	ND	None apparent	Bilateral cavity
P16	17	M	NC	Initial	Mar 2002	None apparent	Bilateral nodular infiltration and cavity in the left lung
P17	17	M	NC	Initial	May 2003	Nephew of patient 14	Wright apical cavity and left lung nodular opacity
P18	21	M	NC	Initial	Jun 2003	Brother of patient 5	Right apical cavity and nodular opacity
P19	42	M	NC	Initial	Jun 2003	No interview (lost case)	Diffuse nodular opacity and multiple cavities
P20	53	M	NC	Follow-up, Oct 2003	Oct 2002	None apparent	Right apical cavities and left lobe infiltrate
P21	ND	M	NC	Initial	Apr 2004	Cousin of patient 14	ND

*PT, previously treated; NC, new case; ND, not determined.

**Table 2:** Laboratory findings and genotyping of multidrug-resistant isolates from 21 tuberculosis patients, Bizerte, Tunisia, 2001–2004*

Patient	Smear result	Resistance pattern†	RFLP‡	Spoligotype	PGG§	Mutational analysis				
						*rpoB*	*katG*	*pncA*	*mutT3*	*Ogt*
P1	+++	HSREZ	11	Haarlem3¶	2	S531L+V610M	S315T	A-11C	L209L	T15S
P2	+	HSREZ	11	Haarlem3	2	S531L+V610M	S315T	A-11C	L209L	T15S
P3	++	HSREZ	12	Haarlem3	2	S531L+V610M	S315T	T11G (L4W)	L209L	T15S
P4	-	HSREZ	11	Haarlem3	2	S531L+V610M	S315T	A-11C	L209L	T15S
P5	-	HSREZ	12	Haarlem3	2	S531L+V610M	S315T	WT	L209L	T15S
P6	+	HSREZ	11	Haarlem3	2	S531L+V610M	S315T	WT	L209L	T15S
P7	-	HSREZ	11	Haarlem3	2	S531L+V610M	S315T	A-11C	L209L	T15S
P8	-	HSRE	11	Haarlem3	2	S531L+V610M	S315T	WT	L209L	T15S
P9	+	HSRE	11	Haarlem3	2	S531L+V610M	S315T	A-11C	L209L	T15S
P10	-	HSREZ	11	Haarlem3	2	S531L+V610M	S315T	WT	L209L	T15S
P11	-	HSREZ	12	Haarlem3	2	S531L+V610M	S315T	WT	L209L	T15S
P12	-	HSREZ	11	Haarlem3	2	S531L+V610M	S315T	A-11C	L209L	T15S
P13	-	HSRE	ND#	Haarlem3	2	S531L+V610M	S315T	WT	L209L	T15S
P14	-	HRZ	11	Haarlem3	2	S531L+V610M	S315T	G insertion (391-392)	L209L	T15S
P15	-	HSREZ	11	Haarlem3	2	S531L+V610M	S315T	A-11C	L209L	T15S
P16	++	HSR	12	Haarlem3	2	S531L+V610M	S315T	T11G (L4W)	L209L	T15S
P17	-	HSREZ	11	Haarlem3	2	S531L+V610M	S315T	G insertion (296-297)	L209L	T15S
P18	-	HSREZ	12	Haarlem3	2	S531L+V610M	S315T	T11G (L4W)	L209L	T15S
P19	-	HSRE	9	Other**	2	ΔN (AAC)519	S315T	G insertion (296-297)	WT	WT
P20	++	HSR	10	Haarlem3	2	S531L	S315	WT	L209L	T15S
P21	++	HR	11	Haarlem3	2	S531L+V610M	S315T	WT	L209L	T15S

*RFLP, restriction fragment length polymorphism; PGG, principal genetic grouping; WT, wild type (identical to strain H37Rv); ND, not determined. †H, isoniazid; S, streptomycin; R, rifampicin; E, ethambutol; Z, pyrazinamide. ‡Number of IS*6110* bands. §Principal genetic grouping according to *gyr*A and *kat*G polymorphisms (11). ¶Absence of spacers 31 and 33–36. #IS*6110* typing was determined by ligation-mediated polymerase chain reaction and the profile was identical to the other outbreak-associated strains. **Absence of spacers 15, 21–24, and 33–36.

**Figure 1:**
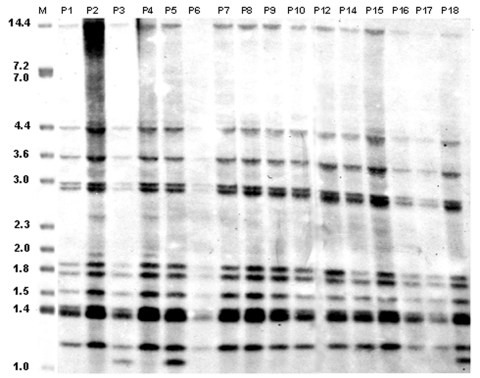
IS*6110* restriction fragment length polymorphism (RFLP) analysis of *Mycobacterium tuberculosis* isolates from 16 patients associated with the multidrug-resistant tuberculosis outbreak, Bizerte, Tunisia, 2001–2004. Lane M, reference strain MTB14323. Values above each well correspond to each patient as identified in Table 1. Values on the left are in kilobases.

**Figure 2:**
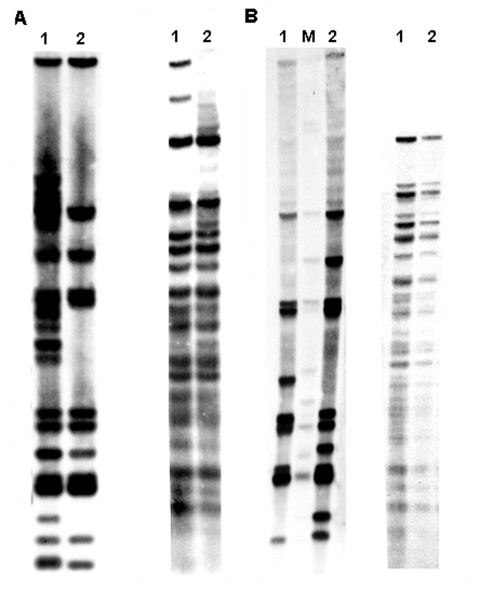
A) IS*6110* restriction fragment length polymorphism (RFLP) analysis (left) and polymorphic GC-rich repetitive sequence (PGRS) typing (right) of patient P11. Lane 1, initial isolate; lane 2, follow-up isolate. B) IS*6110* RFLP (left) and PGRS typing (right) of patient P20 (lane 1) compared with patient P3 (lane 2), a typical outbreak-associated patient. Lane M, reference strain MTB14323.

As indicated in Table 1, with the exception of patient P20, the DNA samples subjected to molecular typing were obtained from the initial isolate of all new patients. RFLP showed that 18 patients had nearly identical IS*6110* profiles (Figures 1 and 2). The predominant profile (occurring in 13 patients) showed 11 bands, while the remaining 5 patients had an additional IS*6110* band. The presence or absence of the additional IS*6110* band was not restricted to new or previously treated patients. The RFLP pattern of patient P11, a new patient, clearly showed a mixture of the 12-band profile and some additional IS*6110* bands (Figure 2A). Typing of his follow-up culture, which was obtained after 6 months of directly observed short-course therapy, as recommended by the World Health Organization, yielded only the 12-band profile (Figure 2A). Laboratory records and epidemiologic data indicate that this patient likely had a dual infection.

The isolate from patient P13 was typed by ligation-mediated PCR. Its profile was identical to the 18 other MDR isolates. Thus 19 patients with MDR-TB could be clustered according to IS*6110*-based typing. Effective epidemiologic links were identified for 9 (47%) patients (Table 1). Another similar RFLP pattern was observed for patient P20. It shows 10 IS*6110* bands (Figure 2B), 9 of which are common to the 12-band RFLP pattern described for the other isolates. The isolate from patient P19 displayed a 9-band IS*6110* profile that was clearly distinct from all the other patients with MDR-TB (data not shown).

With the exception of patient P19, the MDR isolates were identical in their PGRS profile (Figure 2) and spoligotype patterns (Table 2), which is characteristic of the Haarlem3 type (4). Sequence analysis of mutator and drug resistance genes conclusively confirmed that the 19 MDR isolates with nearly identical IS*6110* (both 12- and 11-band profiles) are genetically closely related. They all harbor the L209L, T15S, S531L, and S315T mutations in *mutT3*, *ogt*, *rpoB,* and *katG* genes, respectively (Table 2), whereas *mutT1* and *MutT2* showed a wild type genotype (data not shown). The occurrence of an additional uncommon mutation in the *rpoB* gene (V610M) confirmed the clonality of this MDR Haarlem strain since it was present only in 19 patients with MDR-TB. The variability of resistance to pyrazinamide and the mutational profile within the *pncA* gene (Table 2) strongly suggest that primary transmission from person to person occurred mainly with a strain that was simultaneously resistant to isoniazid and rifampicin.

To extend our analysis of the situation that prevailed in this region, samples from 143 (83%) of 172 patients without MDR strains were spoligotyped. Of these 143 patients, 41 (29%) were female. Overall, 31 (22%) of the 143 patients had Haarlem3 genotype TB. In contrast to the MDR-TB outbreak that involved only men, 6 women had a non-MDR Haarlem3 strain. Aside from the absence of clustering, ligation-mediated PCR typing showed that none of these non-MDR Haarlem3 isolates displayed a profile similar to the 19 MDR isolates involved in the transmission chain. Sequencing of the *rpoB* gene of 10 isolates randomly selected from the 31 non-MDR Haarlem isolates showed the absence of the outbreak-associated mutation V610M. This finding is strongly indicative of a true clonal expansion and a typical MDR-TB outbreak. The W/Beijing type was absent in the analyzed pool of isolates.

## Conclusions

The results indicate that an MDR strain of *M. tuberculosis* has been actively transmitted among 19 HIV-negative male patients in Tunisia. Several observations indicate that this particular Haarlem strain displays increased transmissibility, virulence, or both. First, the outbreak peaked suddenly within a relatively short period of 21 months; 17 new cases (89%) were reported from September 2001 to June 2003. Inspection of the hospital register for 2000 showed only 3 new patients with MDR isolates, including outbreak-associated patients P5 and P7 (Table 1). Second, no epidemiologic links or contact points could be traced for several patients, which suggests that brief exposure would have been sufficient for effective transmission. Because patients with MDR-TB do not respond to treatment, they may serve as constant sources of transmission. Such a situation is likely to have occurred for the patients with established epidemiologic links. Third, the incidence of TB in the region in which the outbreak occurred is not particularly high. Fourth, patients were seronegative for HIV with no history of treatment causing immunosuppression. Fifth, no AIDS-associated TB outbreak that might have increased the adaptability of the strain within the indigenous population had occurred in the region. Sixth, although the Haarlem strain was MDR, it was able to cause an outbreak in those vaccinated with bacille Calmette-Guérin and in persons who were not hospitalized.

Among the identified *M. tuberculosis* strain families (4,5), the W/Beijing type has been associated with outbreaks or microepidemics worldwide (3). The Haarlem strain family appears to be widespread (4), but its ability to cause outbreaks has been reported only twice, once in Argentina (13) and once in the Czech Republic (14). The distinctive feature of the present Haarlem MDR-TB outbreak is its accelerated transmission compared with the first 2 MDR-TB outbreaks.

Alterations within DNA repair genes (mutator genes) are thought to favor the emergence of MDR strains with an increased adaptability (12). In this respect, both W/Beijing and Haarlem strains accumulated mutations within their putative mutator genes. Widespread MDR strains might also benefit from their intrinsic adaptability (15). From an epidemiologic point of view, TB programs must conduct extensive surveillance of MDR strains of *M. tuberculosis* strain families because they might cause serious outbreaks.
